# Age Classification of Rice Seeds in Japan Using Gradient-Boosting and ANFIS Algorithms

**DOI:** 10.3390/s23052828

**Published:** 2023-03-05

**Authors:** Namal Rathnayake, Akira Miyazaki, Tuan Linh Dang, Yukinobu Hoshino

**Affiliations:** 1 School of Systems Engineering, Kochi University of Technology, Kochi 782-8502, Japan; 2 Faculty of Agriculture, Kochi University, Kochi 780-8072, Japan; 3 School of Information and Communications Technology, Hanoi University of Science and Technology, No. 1, Dai Co Viet Road, Hanoi 100000, Vietnam

**Keywords:** Japanese rice, age classification, machine learning, ANFIS, cascaded-ANFIS, XGBoost, CatBoost, LightGBM

## Abstract

The rapidly changing climate affects an extensive spectrum of human-centered environments. The food industry is one of the affected industries due to rapid climate change. Rice is a staple food and an important cultural key point for Japanese people. As Japan is a country in which natural disasters continuously occur, using aged seeds for cultivation has become a regular practice. It is a well-known truth that seed quality and age highly impact germination rate and successful cultivation. However, a considerable research gap exists in the identification of seeds according to age. Hence, this study aims to implement a machine-learning model to identify Japanese rice seeds according to their age. Since agewise datasets are unavailable in the literature, this research implements a novel rice seed dataset with six rice varieties and three age variations. The rice seed dataset was created using a combination of RGB images. Image features were extracted using six feature descriptors. The proposed algorithm used in this study is called Cascaded-ANFIS. A novel structure for this algorithm is proposed in this work, combining several gradient-boosting algorithms such as XGBoost, CatBoost, and LightGBM. The classification was conducted in two steps. First, the seed variety was identified. Then, the age was predicted. As a result, seven classification models were implemented. The performance of the proposed algorithm was evaluated against 13 state-of-the-art algorithms. Overall, the proposed algorithm has a higher accuracy, precision, recall, and F1-score than the others. For the classification of variety, the proposed algorithm scored 0.7697, 0.7949, 0.7707, and 0.7862, respectively. The results of this study confirm that the proposed algorithm can be employed in the successful age classification of seeds.

## 1. Introduction

Japan is a country with a population of 127 million people. For over 2000 years, rice has been a significant staple in Japan. Around 300 of the 40,000 or so distinct types of rice produced worldwide can be found in Japan [1]. A tendency toward sustainable rice production through technical advancements has emerged, particularly in light of the rising scarcity of resources such as water and land. Researchers are driven to find new solutions to the declining or stagnant yields brought on by poor grain quality and rising production costs due to a significant reliance on agricultural inputs.

Nevertheless, despite these limitations, rice output needs to dramatically increase in the following generation to meet the global food demand, especially for the poor. To ensure the food supply and social, economic, and water sustainability of Asia, a region in which rice is deeply culturally ingrained, it is crucial to produce more rice with a restricted or controlled flow of resources [2]. According to previous research, the quality of rice seeds used primarily for rice cultivation is mainly determined by the age of the rice seeds after harvest. Therefore, this research aims to create a method that can more accurately determine or validate the word-of-mouth age of rice seeds, which might be one factor in assessing the overall quality of rice seeds.

Due to its climate and topography, Japan is particularly susceptible to natural disasters, including earthquakes, storms, and flooding. Unfavorable weather conditions may result in crop failure or harvest loss. Hence, using old rice seeds for cultivation is a common practice. By more accurately determining the age of rice seeds, care necessary for treatment might be taken.

In [3], the germination rate behavior of Portulaca oleracea L. seeds was examined in relation to their place of origin, seed maturation period, and seed age. Three geographical areas representing three different climates were used to gather seeds; then, they were cultured under diverse light and temperature conditions. Compared to seeds from the Canadian site, the results showed that seeds from the United Arab Emirates location exhibited less dormancy and germinated more quickly under a wider variety of incubation conditions. At the Canadian location, seed age substantially impacted germination rate but not for seeds from the Egyptian or United Arab Emirates sites. Overall, the study emphasizes the significance of considering environmental factors when determining how seeds adapt to dormancy and germination rate. In [4], the authors looked at how alfalfa’s tolerance to salt during germination rate changed with natural and artificial aging. The findings demonstrated that different aged seed lots had considerably varying salt tolerances and that seed age enhanced the amount of solute leakage that occurred after ingestion.

Moreover, ref. [5] examined the relationship between seed age and seedling vigor and competitiveness in populations of Bromus tectorum, an annual grass found in meadows and sagebrush steppes. When grown in competition, old seeds from the meadow steppe population displayed germination rate delays, which lowered plant growth and biomass, whereas aged seeds from the sagebrush steppe population did not. According to the study, physiological expenses related to seed age may impact aboveground competitive interactions and the relative fitness of older cohorts in the soil seed bank.

Although the relationship between seed age and germination rate is well established in the literature, to our knowledge, no research has been conducted on age-based rice seed categorization. The relevant works on the correlation between germination rate and rice seed age and the classification of seeds are introduced in the following paragraphs. However, these are not restricted to rice.

According to the above-stated literature, there are several reasons why a machine would be necessary to develop a real-time application for classifying rice seed varieties and identifying their harvested age:

- Speed:
  Machines can process and analyze large amounts of data much faster than humans, making it possible to classify and identify rice seed varieties in real-time.
- Accuracy:
  Machines can analyze data with a high level of accuracy, reducing the chances of errors in classification and identification.
- Consistency:
  Machines can perform tasks consistently and accurately, ensuring that the classification and identification of rice seed varieties are consistent across different batches.
- Efficiency:
  Machines can work continuously without breaking, allowing for more efficient and cost-effective data processing.
- Cost:
  Using machines can reduce labor costs, as they can perform tasks that would otherwise require human labor. This can make the development of a real-time application more cost-effective.

Therefore, this study aims to implement a machine learning model that can be employed to develop a real-time application to classify rice seed varieties and identify their harvested age. The following points are introduced as the research outcomes of this study.

1. Implementation of rice seeds dataset based on varieties and harvested age:
  Developing a dataset from scratch when working with AI and machine learning can be challenging, especially if you do not have access to a large and diverse dataset. This is because the performance of a machine learning model is heavily dependent on the quality and quantity of the data on which it is trained. When a dataset is undersized or generated using an artificial method, this can lead to an unsatisfactory performance when using a machine learning algorithm. One solution is to consider whether it is necessary to use a machine learning model. Sometimes, a more straightforward approach, such as a rule-based system or a decision tree, can be used to solve the problem. Here, a dataset was developed from scratch due to the lack of age-based seed image data. This study introduces a rice seed dataset with six different seed varieties and three age categories for the classification task. To our knowledge, this dataset is the only one labeled based on the harvested age of the seeds.
2. Investigation of Red, Green, and Blue (RGB) features for accurate classification: Various features were comprehensively evaluated due to the use of RGB images. The feature descriptors used in this study can be introduced as follows.
  - Color structure;
  - Edge histogram;
  - Region shape;
  - Gray-level co-occurrence matrix (GLCM);
  - Mean value of RGB spaces;
  - Column layout.
3. Implementation of a novel machine-learning model for the cost-effective and efficient identification of the rice seeds’ variety and age: the proposed algorithm is a combination of the gradient-boosting algorithm and cascaded adaptive network-based fuzzy inference system (Cascaded-ANFIS) algorithms. The performances of the proposed algorithm were compared with several feature-based machine learning algorithms.

## 2. Related Works

In-depth research on the correlation between germination rate and rice seed age was conducted by Jones et al. in 1926 [6]. For the majority of rice types, seed age was suggested to be inversely correlated with the germination rate. Eight different types of rice were employed in the study, and age intervals of six years were investigated. According to a survey, the association between germination rate and wheat seed age was the same as in the prior study. According to them, the germination rate declines at a rate of 0.243% h^−1^ as people age [7]. Canola seeds were studied by Yun et al. to determine how much seed aging affects the germination rate [8]. They also showed that older seeds have a lower germination rate than fresh ones.

Additionally, Ibrahim et al. in 2013 [9] and Tabatabaei in 2014 [10] reported results from two different studies on seed germination rate with aging and reached the same conclusions. These signs play a significant role in the age-based categorization of seeds. Wu and Tsai presented a leaf image noise reduction. They were able to achieve 92.13% accuracy using background removal and ROI extraction approaches as the innovative forms of implementation [11].

For various tasks assessing food quality, machine vision systems have been developed [12,13,14,15]. Research has concentrated on fusing image analysis with machine learning techniques to create new automatic inspection and certification approaches. The quality control or cultivar categorization tasks primarily examined in [16] for rice seeds (polished) are pertinent to the work presented here. Y. Ogawa provides a thorough overview of computer vision methods, measurements of physical properties, chemical content, and distributions of rice grains for seed quality management in [16].

Using an automated machine vision system to classify rice seeds often involves many steps. Collecting picture data and feature extraction are among the most crucial examples. Morphological, color, and textural qualities, individually or in combination, are frequently used in appearance-based techniques. Lai et al. [17] proposed using image analysis to detect the physical parameters of and manually categorize wheat grains, a proposal that dates back to 1986. Two-dimensional image analysis is used by Sakai et al. [18] to manually categorize four different species of polished rice grains by extracting their dimensions and form parameters. It is common practice to extract the form descriptors from the seed samples and train classifiers such as random forests (RF) [19], neural networks (NN) [20], or a Cubic B-Splines shape model [21].

In contrast to characteristics that are more frequently employed in the literature, such as the chaff tip and depth of concavities of rice kernels, Huang et al. [22] conducted an extensive examination of shape descriptors. While just three kinds of rice were evaluated, their study demonstrates encouraging results in the separation of superficially similar species. With a standard derivation of 7.0%, Kuo et al. [23] investigated 30 types of rice seeds using sparse representation classification with an accuracy of 89.1%. The bulk of the literature employs a small number of species, which the writers briefly acknowledged. However, they did not show how this may affect the reader’s capacity to discriminate between different species. They used a systematic approach, concentrating on the grains’ specific regions of interest.

HSI approaches have recently been used in food and agricultural engineering. Wang et al. [24], using VIS/NIR spectral data, distinguished three different rice cultivars. The authors combined several features from the obtained HSI pictures, including the degree of chalkiness, form features, and spectral properties. Principle component analysis (PCA) was utilized to decrease the dimensionality of the spectral data. The principal components were then used to train an artificial neural network, yielding a classification accuracy of 94.45%. The authors of [25] found a practical way to monitor the nitrogen status in rice using a combination of the least squares support vector machine (LS-SVM) regression algorithm and VIS/NIR spectroscopy within a range of 325–1075 nm. Four rice seed varieties were recently identified using an HSI technique in [26]. The authors [26] achieved up to 100% accuracy in their findings using a random forest (RF) classifier and the whole spectral range of their system, 1039–1612 nm. It is uncertain how the inter/intra-class changed over the four cultivars in [26] because they were crossbred with different species.

The studies in [27,28] investigated several feature combination techniques to find the best feature combination. When combining spectral, texture characteristics, and morphology, the best accuracy (91.67%) was attained in [28]. Using a dataset containing six different rice seed types and a mix of spectral and spatial information, the authors of [27] reported a classification accuracy of 84%.

The previous studies on this subject do not provide sufficient information on the age classification of seeds. Therefore, this study is novel to the best of the authors’ knowledge. There are several methods of seed variety classification based on hyperspectral images. However, a hyperspectral dataset does not provide a convenient environment to implement a system that farmers and other interested parties can use. Hence, the main focus of this study is to implement a comparatively effective and efficient rice seed classification system based on harvested age.

## 3. Methodology

### 3.1. Dataset Construction and Preprocessing

**Figure 1:**
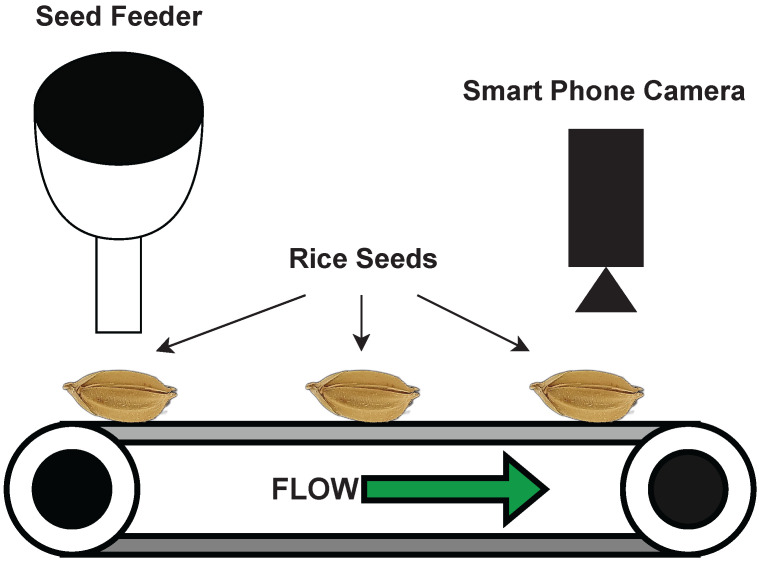
Rice seeds dataset construction setup.

The rice seed dataset was newly constructed. This study was conducted with six rice varieties of different harvest ages. Initially, the rice seeds were collected with the help of Prof Akira Miyazaki from the Agriculture Faculty at Kochi University, Japan. Figure 1 shows that the dataset was constructed using a conveyer belt setup. The ultimate objective of this study is to implement a mobile application that can be used in real-time with a smartphone camera. Therefore, as shown in the figure, a smartphone camera was used for data acquisition.

The smartphone that we used was Xiaomi 11T Pro. The macro-lens was used in the camera configuration to obtain feature-rich images. The specifications of the macro-lens are as follows: a 5 Megapixel 1/5′ sensor with an f/2.4 aperture lens. A controller was developed to synchronize the conveyor belt movements and camera shuttle speed. The images were obtained at five-second intervals. Each time, the conveyor belt stopped and obtained a macro-image of the rice seed. The images were saved in JPEG format.

**Figure 2:**
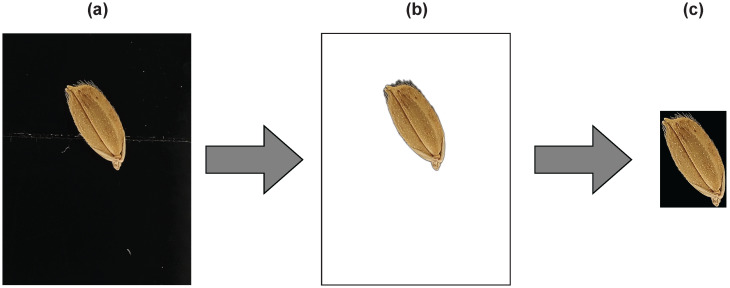
Image preprocessing steps. (**a**) The images were captured using a smartphone camera. (**b**) The backgrounds were removed from the raw dataset. (**c**) Seeds were cropped and isolated.

The dataset was preprocessed in three steps: (a) the images were captured using the smartphone camera; (b) the backgrounds were removed from the raw dataset; (c) seeds were cropped and isolated. The background was removed using the *rembg* library in python, and segmentation was carried out using the OpenCV4 platform. The outputs of each process are shown in Figure 2.

**Figure 3:**
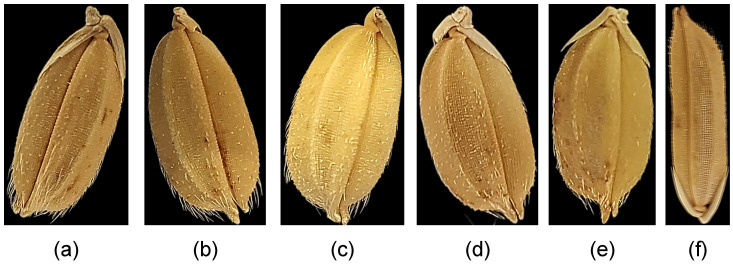
Rice Varieties (harvested in 2012). (**a**) Akitakomachi. (**b**) Fusaotome (**c**) Hatsuboshi. (**d**) Koshihikari. (**e**) Okiniiri. (**f**) Yang Dao-8.

Table 1 shows the completed dataset’s descriptive information. Six rice varieties were used to develop the dataset, namely, Akitakomachi, Fusaotome, Hatsuboshi, Koshihikari, Okiniiri, and Yang DAO-8 (Figure 3). These rice varieties originated in Japan, except Yang DAO-8. Yang DAO-8 originated in China. The harvest ages of these rice samples were 2012, 2016, and 2020. However, Okiniiri seeds comprised only samples from 2012 and 2016, while Yang DAO-8 contained samples from 2012 and 2020. Hence, the total number of classes could be calculated as 16. The completed dataset was uploaded and is publicly available in the Kaggle data repository under Japanese rice seeds agewise classification.

The dataset was divided at a ratio of 7:3 for training and testing, and the same datasets were employed for all algorithms used in this study.

### 3.2. Feature Extraction

The main component in classifier implementation is feature extraction. As a result, six feature descriptors were used to extract various features from the rice seed dataset. Each of these feature extraction techniques is briefly described in this section. The Color Structure descriptor is the first technique. Although it is based on histogram equalization, it aims to differentiate localized color differences for each color and provides a comprehensive explanation [29]. The Region Shape is the following feature description. Due to the inherent challenges in depicting forms, the shape features are less developed than their color and textural equivalents [30]. It is not feasible to precisely segment an image into meaningful regions using low-level features because of the variety of ways in which a 3D object can be projected into 2D shapes, the complexity of each object’s shape, and the presence of shadows, occlusions, non-uniform illumination, and varying surface reflectivity. As a result, the third feature extraction technique employed the column layout feature descriptor.

The edge histogram (EDH) descriptor was used to show how local edges are distributed throughout images [31]. As a result, this study’s fourth feature extraction technique was the EDH descriptor. The histogram was used to describe edges, a crucial aspect for visualizing picture data. The EDH-described characteristics of a picture cannot be replicated by the uniform color histogram and texture features [32,33]. The gray level co-occurrence matrix is the fifth characteristic descriptor (GLCM). Given a picture made up of pixels, each with a certain intensity, it calculates how frequently particular pairings of gray levels co-occur in an image or part of an image (a specific gray level). The change in intensity at the pixel of interest is measured using the GLCM contents in texture feature computations [34]. Moreover, the sixth feature descriptor was the mean values of red, green, and blue channels.

### 3.3. Machine Learning Algorithm Development

#### 3.3.1. Gradient-Boosting Algorithms

Gradient-boosting algorithms are used in most literature studies due to their easy implementation, low computational cost and efficiency. This study proposes a novel machine-learning method for rice seed classification, and gradient-boosting algorithms are one of its key components. Gradient-boosting algorithms are based on the suspicion that the overall prediction error is minimized when previous models are combined with the best possible forthcoming model. Setting the expected outcomes for this following model is crucial to minimizing errors. Each case’s target outcome will differ depending on how changing a case’s forecast impacts the overall prediction error. This technique is referred to as “gradient boosting” because target outcomes are defined for each case depending on the gradient of the inaccuracy of the prediction. Each new model advances in a way that minimizes prediction error in the potential predictions for each training instance [35].

- XGBoost: eXtreme Gradient Boosting.
  Chen et al. [36] invented the XGBoost algorithm. Gradient-boosting machines are used in a novel and extensible way that has been found to increase the computational efficiency of boosted tree algorithms. They were developed specifically to boost model performance and computational effectiveness.
  In an ensemble strategy known as “boosting”, adding more models fixes the errors introduced by previous models. The gradient-boosting approach involves the creation of new models that predict the residuals of previous models, which are then integrated to obtain the conclusive prediction. The model addition process is repeated until there is no observable improvement. A gradient descent method reduces the loss when adding new models.
  A total of 17 of the 29 machine learning (ML) projects posted on Kaggle were successfully completed by XGBoost by 2015. Speed was significantly boosted by using many CPU cores, reducing the look-up times of individual trees created with XGBoost. This method was constructed in R and Python using the SciKit-Learn [37] package and uses unique regularization approaches.
- CatBoost: Categorical Boosting.
  Diverse characteristics, noisy data, and complex connections can be dealt with using a powerful machine-learning technique called gradient boosting. In 2017, CatBoost, a machine learning method based on gradient-boosting decision trees (GBDT), was introduced by Yandex engineers [38]. CatBoost has the following advantages over other GBDT algorithms:
  1. The algorithm effectively handles category features. Using traditional GBDT methods, categorical traits can be substituted by suitable average label values.
  2. In CatBoost, many category traits are blended. CatBoost uses a greedy approach to integrate all categorical features from the dataset with all categorical traits and combinations in the current tree.
  3. CatBoost can be used to alleviate gradient bias. Each iteration of GBDT generates a weak learner, and each learner is taught using the gradient of the preceding learner. The total findings from each learner’s categorization comprise the output [39].
- LightGBM: Light Gradient Boosting.
  The LightGBM [40] algorithm from Microsoft is an open-source GBDT. The histogram-based approach provides the foundation for the parallel voting decision tree technique, which speeds up training, uses less memory, and integrates complex network connections to maximize parallel learning [41,42]. In each iteration, the local voting decision is made, selecting the top k characteristics and the global voting choice to receive the top attributes. The training data are distributed among many computers. LightGBM uses the leaf-wise method to determine which leaf has the most significant splitter gain.

#### 3.3.2. Adaptive Network-based Fuzzy Inference System (ANFIS)

A multi-layer adaptive network-based fuzzy inference system called ANFIS was suggested by Jang [43]. When learning and fine-tuning fuzzy inference system (FIS) parameters using a hybrid learning mode, an ANFIS consists of five layers that implement various node functions. The following parameters are updated, and the errors are transferred to the backward pass using the least squared error estimation approach in the forward pass with fixed premise parameters. While fixing the subsequent parameters, the backward pass changes the premise parameters using the gradient descent method. The assumption and associated parameters for membership functions (MF) and fussy inference system (FIS) will be revealed by repeatedly performing forward and backward passes. In automation control [44] and other domains, ANIFS is frequently used.

### 3.4. Cascaded-ANFIS

Cascaded-ANFIS is an extension of the ANFIS algorithm. The Cascaded-ANFIS was introduced in 2021 and showed several benefits compared to the traditional ANFIS algorithm. ANFIS has two significant limitations, such as the curse of dimensionality and higher computational power consumption. The Cascaded-ANFIS removes these limitations using a simple ANFIS algorithm in different configurations.

**Figure 4:**
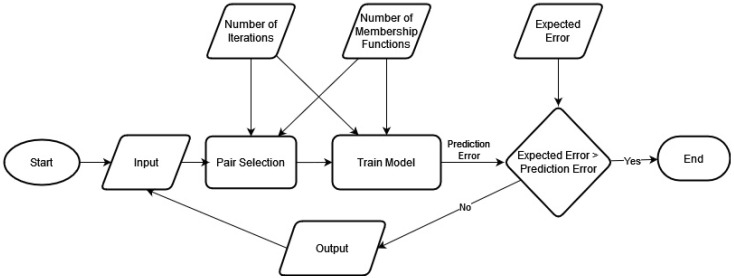
Construction of the Cascaded-ANFIS algorithm.

Figure 4 illustrates the Cascaded-ANFIS algorithm’s creation.

As shown in the figure, there are two primary components: (1) pair selection and (2) train model. Generally, the Cascaded-ANFIS algorithm selects the best pairs and trains them individually using a two-input–one-output ANFIS model. Then, the output of each node is transferred to the next level as the input.

The pair selection module uses the sequential feature selection (SFS) procedure. The novel aspect of the method is identifying the best fit for each input variable using a two-input–one-output ANFIS model. A nested loop cycles through all potential pair combinations to achieve this. The two-input ANFIS model is then used with these as inputs. Then, the root means square error (RMSE) is calculated, recorded, and compared to the previous RMSE. Finding the lowest RMSE value at the end of the second loop will reveal the matched pair. The training phase can start after the pairings are chosen.

In the training instance, a two-input ANFIS model is also used. The input can be directly sent to the ANFIS module, which can produce current outputs and RMSE for each data pair, as the input variables are paired with the best match from the previous module. There is also a target error in place at this point. As a result, the goal error and RMSE are contrasted. If the desired error is achieved, the process may be terminated. If not, the algorithm moves on to the second iteration.

Rathnayake et al. [45,46] provide further information and technical specifics regarding Cascaded-ANFIS. The novel Cascaded-ANFIS method is capable of handling computational complexity with ease. The distinctive methods created by Cascaded-ANFIS can also manage noisy datasets.

### 3.5. Proposed Approach

**Figure 5:**
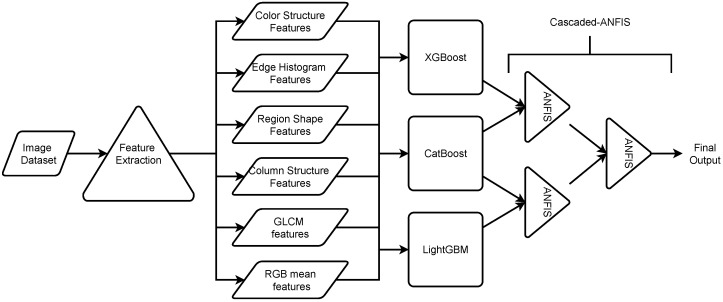
Construction of the novel proposed algorithm.

One of the crucial benefits of the Cascaded-ANFIS algorithm is that it can be restructured according to the problem statement. The present study is based on image data, and features are extracted from the images. This method provides an extensive number of feature dimensions. Therefore, this study proposes combining selected gradient-boosting algorithms (XGBoost, CatBoost, and LightGBM) in a Cascaded-ANFIS structure. The proposed approach is illustrated in Figure 5.

As shown in the figure, the image data extracted features employing six feature descriptors. The total number of features that were used in this study is 159. Then, the extracted features were fed to the gradient-boosting algorithms to predict the output. These outputs were then used to train the Cascaded-ANFIS algorithm. The figure shows that the pairs were pre-assigned as XGBoost-CatBoost and CatBoost-LightGBM. This combination was selected based on the testing results of all pairs. The proposed novel structure of the Cascaded-ANFIS algorithm has two levels.

The parameter tuning of each algorithm was performed using the GridSearchCV method of the sci-kit-learn library. The tuned parameters and their values are presented in Table 2 below. This study was conducted purely in a CPU-based environment. The experimental platform information is presented in Table 3.

## 4. Experimental Results

### 4.1. Evaluation Criteria

Using a confusion matrix, the proposed model’s performance was examined. Classification matrices were computed and illustrated in Equations (1)–(4) to comprehend the confusion matrix.

(1) $$Accuracy_{Avg}=\frac{\sum_{i=1}^{l}\frac{tp_{i}+tp_{i}}{tp_{i}+fn_{i}+fp_{i}+tn_{i}}}{l}$$

(2) $$Precision_{\mu }=\frac{\sum_{i=1}^{l}tp_{i}}{\sum_{i=1}^{l}\left(tp_{i}+fp_{i}\right)}$$

(3) $$Recall_{\mu }=\frac{\sum_{i=1}^{l}tp_{i}}{\sum_{i=1}^{l}\left(tp_{i}+fn_{i}\right)}$$

(4) $$F1\mathrm{-S}core_{\mu }=\frac{\left(\beta^{2}+1\right)Precision_{\mu }Recall_{\mu }}{\beta^{2}Precision_{\mu }+Recall_{\mu }}$$

True Positive, False Positive, True Negative and False Negative are denoted as $tp_{i},fp_{i},tn_{i},$ and $fn_{i}$, respectively. Moreover, *l* and μ indicate the number of classes and micro-average. When the problem is multiclass, each of these factors offers essential information about the effectiveness of the classification [47].

Moreover, the experiment was conducted in several steps. The planned identification process has two steps: (1) identifying the rice variety; (2) identifying the rice seed age. Hence, this study contains seven classification tasks (i.e., classification between the six rice varieties and age classification models of six rice varieties).

Nevertheless, the performances of the proposed algorithm were comprehensively evaluated with 13 other algorithms. These are as follows.

1. Nearest neighbors;
2. Linear support vector machines (Linear SVM);
3. Radial basis function kernel SVM (RBF SVM);
4. Gaussian process;
5. Decision tree;
6. Random forest;
7. Neural net;
8. Adaptive boosting (AdaBoost);
9. Naive Bayes;
10. Quadratic discriminant analysis (QDA);
11. XGBoost;
12. CatBoost;
13. LightGBM.

### 4.2. Rice Variety Classification

Table 4 shows the performance in terms of rice variety classification. The table shows that the proposed model achieved a maximum accuracy, precision, recall, and F1-score of 0.7697, 0.7949, 0.7707, and 0.7862.

### 4.3. Age Classification of Each Variety

Once the classification of the rice variety is completed, the age of the rice has to be identified. Therefore, several classifiers were trained to evaluate the classification results.

#### 4.3.1. Akitakomachi

Table 5 shows the Akitakomachi rice seed age classification. The results are promising. The proposed model performed better than other algorithms. However, the XGBoost algorithm performance was significantly similar to that of the proposed model. The table shows that the proposed model achieved a maximum accuracy, precision, recall, and F1-score of 0.7551, 0.7579, 0.7552, and 0.7556.

#### 4.3.2. Fusaotome

Table 6 shows the Fusaotome rice seed age classification. The results were the same as those of the Akitakomachi. The proposed model performed better than other algorithms. However, the CatBoost algorithm performance was significantly similar to that of the proposed model. The table shows that the proposed model achieved a maximum accuracy, precision, recall, and F1-score of 0.8612, 0.8720, 0.8618, and 0.8616.

#### 4.3.3. Hatsuboshi

Table 7 shows the Hatsuboshi rice seed age classification. The classification results indicated that the neural nets achieved better results than other algorithms. The proposed model shows the second-best performance. However, the results were significantly similar to those of the neural nets. The table shows that the proposed model achieved a maximum accuracy, precision, recall, and F1-score of 0.7989, 0.8193, 0.7976, and 0.7912.

#### 4.3.4. Koshihikari

Table 8 shows the Koshihikari rice seed age classification. The results are promising. The proposed model performed better than other algorithms. However, the XGBoost algorithm performance was significantly similar to that of the proposed model. The table shows that the proposed model achieved a maximum accuracy, precision, recall, and F1-score of 0.8815, 0.8806, 0.8810, and 0.8693.

#### 4.3.5. Okiniiri

Table 9 shows the Okiniiri rice seed age classification. The proposed model performance did not achieve the best results. The LightGBM model showed the best results in terms of Okiniiri rice seed age classification. However, the proposed algorithm’s performance was significantly similar to that of the LightGBM model. The table shows that the proposed model achieved a maximum accuracy, precision, recall, and F1-score of 0.9512, 0.9521, 0.9514, and 0.9512, while LightGBM poses 0.9583, 0.9585, 0.9583, and 0.9583, respectively.

#### 4.3.6. Yang DAO-8

Table 10 shows the Yang DAO-8 rice seed age classification. The proposed algorithm achieved the best results in terms accuracy and recall, and F1-score, i.e., 0.7639, 0.7665, and 0.7479. The precision of the proposed algorithm is second to the LightGBM, which is 0.8196, while the proposed algorithm achieved a score of 0.8062. The difference between the precision values is 0.01. Hence, the proposed algorithm can also be selected as the best approach for this classification task.

## 5. Discussion

Most of the experiment results showed that the proposed algorithm outperformed the other algorithms used in this study. In terms of rice variety classification, the proposed algorithm showed an accuracy of 0.7697, while the second-best algorithm XGBoost achieved an accuracy of 0.7633. The LightGBM and CatBoost algorithm achieved 0.7366 and 0.6288 accuracies, respectively. The rice varieties used in this study are very similar in shape, except for the Yang Dao-8. As shown in Figure 3, Akitakomachi, Fusaotome, Hatuboshi, Koshihikari, and Okiniiri rice seeds are similar in shape. However, the Yang Dao-8 rice seed is longer than the other seeds. This structure similarity impacted the rice seeds’ classification due to the use of Edge Histogram Features.

The results of the age classification of each variety mainly depend on the texture changes due to the aging of the seeds. The Akitakomachi, Fusaotome, Koshihikari, and Yang Dao-8 seed age-based classification was dominated by the proposed model, which achieved the best accuracies. However, the neural nets gave the best results for the Hatsuboshi rice seed age classification, with an accuracy of 0.8167, while the proposed model showed a 0.7989 accuracy. The difference between the accuracies of neural nets and the proposed model is 0.0178, which is significantly small. The best Okiniiri rice age classification accuracy was achieved by the LightGBM model, while the proposed model showed the second-best results, with accuracies of 0.9583 and 0.9512, respectively. The difference between these two models is 0.0071.

Overall, it can be stated that the proposed algorithm showed the best results in all experiments. There are several reasons for the proposed algorithm’s obtaining the best results. Considering each experiment and gradient-boosting model result, it can be seen that each experiment has a different best form: XGBoost, CatBoost and LightGBM. For example, the XGBoost algorithm performed well in the Koshihikari age classification, while in the Okiniiri classification, the LightGBM outperformed XGBoost. As shown in Figure 5, the proposed algorithm is a combination of these three gradient-boosting algorithm results. In other words, the Cascaded-ANFIS section of the proposed algorithm does not depend on the features of the image dataset but on the results of the gradient-boosting models. Therefore, the fuzzy-based ANFIS algorithm calculates the reasoning by mixing the three gradient-boosting models to enhance the performance. This can be clearly seen in each experiment as the algorithm outperformed the gradient-boosting model accuracies. The two-input–one-output ANFIS models generate precise membership functions to deal with the outputs of the gradient-boosting algorithms and enhance the overall accuracy.

## 6. Conclusions

Identifying seeds by their age is a challenging task. To the best of our knowledge, there are zero studies in the literature on this subject. Therefore, this study aimed to design and develop a machine learning algorithm to classify seeds by age. Due to the lack of data availability, the main objective of this work was to construct a novel dataset using six varieties of Japanese rice. Each rice variety included three harvesting ages, except for Okiniiri and Yang DAO-8. The harvested ages of these rice varieties were 2012, 2016, and 2020.

The rice seed dataset was implemented by constructing a conveyor belt system to automatically acquire the seed images. A smartphone camera with a macro-lens captured many surface features and the system’s real-time flexibility. The dataset was divided into a training set and a testing set at a ratio of 7:3, and the same datasets were used to model the classifiers. Six feature-extractors were used to find the critical points of the RGB image dataset. The features were selected based on the success rate and popularity of previous research.

The proposed algorithm is a combination of four unique machine-learning algorithms. XGBoost, CatBoost, and LightGBM gradient-boosting algorithms were the base classifiers of the proposed algorithm, and the secondary output was tuned using the Cascaded-ANFIS algorithm. The Cascaded-ANFIS can change the structure depending on the problem statement. This study has a high feature dimension, and using the Cascaded-ANFIS as the base classifier could rapidly increase the time consumption and computational power. Therefore, gradient-boosting algorithms were initially appointed to predict the classification outputs, while the Cascaded-ANFIS evaluated the base results.

The experiment was conducted using two steps: classification based on the rice variety and identifying the age of the seed. Since six rice varieties were available, seven classifiers were trained accordingly. Each classification task was evaluated using the confusion matrix parameters: accuracy, precision, recall, and F1-score. Moreover, the performances of the proposed algorithm were comprehensively assessed by training 13 other machine learning algorithms. The results indicate that the proposed algorithm is more capable of identifying the seed variety and age. Although other algorithms obtained better results for some occurrences, the differences in the results between other algorithms and the proposed algorithm were insignificant.

According to the results of this study, the proposed algorithm can identify the variety of the seed and age with higher efficiency and effectiveness. The algorithms were trained using only the CPU power. Therefore, this study can be considered as a solution to replace black box algorithms that require higher complexity and increased power consumption. How to implement the machine learning model as a server and introduce it as a mobile phone application can comprise future study objectives. Improving the dataset density could also be a future research goal.

## Figures and Tables

**Table 1:** Rice seed dataset sample count.

Rice Variety	2012	2016	2020
Akitakomachi	306	279	366
Fusaotome	325	416	482
Hatsuboshi	376	495	499
Koshihikari	340	348	509
Okiniiri	345	292	N/A
Yang DAO-8	343	N/A	261

**Table 2:** Machine learning algorithm parameters tuned using the GridSearchCV.

Algorithm	Parameter	Value
XGBOOST	objective	multi:softmax
	colsample_bytree	0.5
	learning_rate	0.3
	max_depth	6
	Alpha	10
	n_estimators	750
	subsample	0.7
CatBoost	Iterations	90
	learning_rate	0.04
	eval_metric	MultiClass
	sampling_frequency	PerTree
	penalties_coefficient	1
	max_leaves	64
	permutation_count	4
	Depth	4
LightGBM	num_leaves	31
	objective	binary
	learning_rate	0.1
	boosting_type	gbdt
Cascaded-ANFIS	Membership_Function	Gaussian
	Number_of_MFs	3
	Number_of_Inputs	2
	Iterations	100

**Table 3:** Operating system specifications.

Processor	Intel(R) Core(TM) i9-10900K
CPU Frequency	3.70GHz
RAM	64.0 GB (63.9 GB usable)
Operating System	Windows 10 Education Version 22H2
System Type	64-bit, x64-based processor
Programming Language	Python 3
Programming Environment	Anaconda

**Table 4:** Rice seed variety classification results.

Algorithm	Accuracy	Precision μ	Recall μ	F1-Score μ
Nearest Neighbors	0.5011	0.5789	0.5102	0.5316
Linear SVM	0.7133	0.7542	0.7218	0.7338
RBF SVM	0.1333	0.0222	0.1667	0.0392
Gaussian Process	0.1378	0.1890	0.1722	0.0501
Decision Tree	0.4922	0.5579	0.5074	0.4871
Random Forest	0.3356	0.4797	0.3324	0.3303
Neural Net	0.7478	0.7686	0.7546	0.7601
AdaBoost	0.5344	0.5793	0.5546	0.5622
Naive Bayes	0.4122	0.5024	0.4435	0.3383
QDA	0.4022	0.5099	0.399	0.3854
XGBoost	0.7633	0.7918	0.7685	0.7769
CatBoost	0.6288	0.6818	0.6351	0.6501
LightGBM	0.7366	0.7716	0.7412	0.7517
**Proposed model**	**0.7697**	**0.7949**	**0.7707**	**0.7862**

**Table 5:** Age classification of Akitakomachi rice seeds.

Algorithm	Accuracy	Precision μ	Recall μ	F1-Score μ
Nearest Neighbors	0.5556	0.5595	0.5556	0.5569
Linear SVM	0.6667	0.6676	0.6667	0.6651
RBF SVM	0.3333	0.1111	0.3333	0.1667
Gaussian Process	0.3500	0.4470	0.3500	0.2012
Decision Tree	0.6500	0.6588	0.6500	0.6498
Random Forest	0.5500	0.5595	0.5500	0.5490
Neural Net	0.6667	0.6682	0.6667	0.6656
AdaBoost	0.6333	0.6297	0.6333	0.6283
Naive Bayes	0.5444	0.5918	0.5444	0.5234
QDA	0.4444	0.6389	0.4444	0.3605
XGBoost	0.7500	0.7507	0.7500	0.7483
CatBoost	0.6722	0.6728	0.6722	0.6686
LightGBM	0.7111	0.7124	0.7111	0.7087
**Proposed model**	**0.7551**	**0.7579**	**0.7552**	**0.7556**

**Table 6:** Age classification of Fusaotome rice seeds.

Algorithm	Accuracy	Precision μ	Recall μ	F1-Score μ
Nearest Neighbors	0.7278	0.7232	0.7278	0.7224
Linear SVM	0.8278	0.8275	0.8278	0.8266
RBF SVM	0.3333	0.1111	0.3333	0.1667
Gaussian Process	0.3444	0.7790	0.3444	0.1899
Decision Tree	0.6556	0.6510	0.6556	0.6505
Random Forest	0.6222	0.6617	0.6222	0.6157
Neural Net	0.8222	0.8212	0.8222	0.8209
AdaBoost	0.7944	0.7960	0.7944	0.7930
Naive Bayes	0.5556	0.7108	0.5556	0.4528
QDA	0.5056	0.7193	0.5056	0.4715
XGBoost	0.8611	0.8622	0.8611	0.8608
CatBoost	0.8611	0.8643	0.8611	0.8615
LightGBM	0.8556	0.8563	0.8556	0.8556
**Proposed model**	**0.8612**	**0.8720**	**0.8618**	**0.8616**

**Table 7:** Age classification of Hatsuboshi rice seeds.

Algorithm	Accuracy	Precision μ	Recall μ	F1-Score μ
Nearest Neighbors	0.6167	0.6181	0.6167	0.5947
Linear SVM	0.8056	0.8213	0.8056	0.7957
RBF SVM	0.3333	0.1111	0.3333	0.1667
Gaussian Process	0.8111	0.8232	0.8111	0.8031
Decision Tree	0.7056	0.7084	0.7056	0.6929
Random Forest	0.5722	0.6676	0.5722	0.5174
**Neural Net**	**0.8167**	**0.8256**	**0.8167**	**0.8090**
AdaBoost	0.7556	0.7636	0.7556	0.7577
Naive Bayes	0.6000	0.6178	0.6000	0.5562
QDA	0.4611	0.6769	0.4611	0.3608
XGBoost	0.7944	0.8172	0.7944	0.7838
CatBoost	0.7611	0.7906	0.7611	0.7458
LightGBM	0.7778	0.7902	0.7778	0.7650
Proposed model	0.7989	0.8193	0.7976	0.7912

**Table 8:** Age classification of Koshihikari rice seeds.

Algorithm	Accuracy	Precision μ	Recall μ	F1-Score μ
Nearest Neighbors	0.6000	0.5960	0.6000	0.5882
Linear SVM	0.8167	0.8131	0.8167	0.8118
RBF SVM	0.3333	0.1111	0.3333	0.1667
Gaussian Process	0.8278	0.8380	0.8278	0.8227
Decision Tree	0.6833	0.6759	0.6833	0.6772
Random Forest	0.6167	0.6681	0.6167	0.5673
Neural Net	0.8556	0.8590	0.8556	0.8525
AdaBoost	0.7278	0.7332	0.7278	0.7302
Naive Bayes	0.4611	0.5158	0.4611	0.3832
QDA	0.5556	0.3812	0.5556	0.4479
XGBoost	0.8722	0.8729	0.8722	0.8692
CatBoost	0.7278	0.7451	0.7278	0.6904
LightGBM	0.8556	0.8551	0.8556	0.8511
**Proposed model**	**0.8815**	**0.8806**	**0.8810**	**0.8693**

**Table 9:** Age classification of Okiniiri rice seeds.

Algorithm	Accuracy	Precision μ	Recall μ	F1-Score μ
Nearest Neighbors	0.8917	0.9053	0.8917	0.8907
Linear SVM	0.9500	0.9500	0.9500	0.9500
RBF SVM	0.5000	0.2500	0.5000	0.3333
Gaussian Process	0.8917	0.9053	0.8917	0.8907
Decision Tree	0.9000	0.9000	0.9000	0.9000
Random Forest	0.9000	0.9072	0.9000	0.8996
Neural Net	0.9500	0.9520	0.9500	0.9499
AdaBoost	0.9583	0.9585	0.9583	0.9583
Naive Bayes	0.8917	0.9053	0.8917	0.8907
QDA	0.5083	0.7521	0.5083	0.3516
XGBoost	0.9500	0.9505	0.9500	0.9500
CatBoost	0.9417	0.9428	0.9417	0.9416
**LightGBM**	**0.9583**	**0.9585**	**0.9583**	**0.9583**
Proposed model	0.9512	0.9521	0.9514	0.9512

**Table 10:** Age classification of Yang DAO-8 rice seeds.

Algorithm	Accuracy	Precision μ	Recall μ	F1-Score μ
Nearest Neighbors	0.6167	0.6630	0.6167	0.5873
Linear SVM	0.7250	0.7723	0.7250	0.7125
RBF SVM	0.5000	0.2500	0.5000	0.3333
Gaussian Process	0.6500	0.6918	0.6500	0.6298
Decision Tree	0.6333	0.6778	0.6333	0.6089
Random Forest	0.5417	0.6502	0.5417	0.4406
Neural Net	0.7500	0.7813	0.7500	0.7429
AdaBoost	0.7083	0.7718	0.7083	0.6902
Naive Bayes	0.5167	0.5404	0.5167	0.4334
QDA	0.5000	0.2500	0.5000	0.3333
XGBoost	0.7500	0.7976	0.7500	0.7396
CatBoost	0.6750	0.7824	0.6750	0.6409
**LightGBM**	0.7500	**0.8196**	0.7500	0.7356
**Proposed model**	**0.7639**	0.8062	**0.7665**	**0.7479**

## Data Availability

The dataset that was used in this study is publicly available in the Kaggle data repository. (https://www.kaggle.com/datasets/namalrathnayake1990/japanese-rice-seeds-agewise-classification (accessed on 15 January 2023)).

## Abbreviations

The following abbreviations are used in this manuscript:

AdaBoost	adaptive boosting
CPU	central processing unit
CatBoost	categorical boosting
Cascaded-ANFIS	cascaded adaptive network-based fuzzy inference system
DOAJ	directory of open access journals
FIS	fuzzy inference system
GBDT	gradient-boosting decision trees
GLCM	gray level co-occurrence matrix
HSI	hyper-spectral images
XGBoost	eXtreme gradient boosting
LS-SVM	least squares support vector machines
LightGBM	light gradient boosting
MF	membership function
MDPI	multidisciplinary digital publishing institute
ML	machine learning
NN	neural networks
PCA	principle component analysis
RGB	red, green, and blue
ROI	region of interest
RF	random forests
RBF	radial basis function
QDA	quadratic discriminant analysis
